# Factors Associated with the Level of Knowledge about Biosafety against COVID-19 in Peruvian Dental Students: A Cross-Sectional Study under a Multivariable Regression Model

**DOI:** 10.3390/ijerph20115938

**Published:** 2023-05-24

**Authors:** John Santome-Pariona, Gissela Briceño-Vergel, Nancy Córdova-Limaylla, Marysela Ladera-Castañeda, José Huamani-Echaccaya, Rita Tolmos-Valdivia, Juan Huamani-Cantoral, Fredy Solís-Dante, Luis Cervantes-Ganoza, César Cayo-Rojas

**Affiliations:** 1 School of Stomatology, Universidad Privada San Juan Bautista, Ica 11002, Peru; john.santome@upsjb.edu.pe (J.S.-P.); gissela.briceno@upsjb.edu.pe (G.B.-V.); nancye.cordova@upsjb.edu.pe (N.C.-L.); jose.huamani@upsjb.edu.pe (J.H.-E.); rita.tolmos@upsjb.edu.pe (R.T.-V.); juane.huamani@upsjb.edu.pe (J.H.-C.); 2 Research Team “Salud Pública–Salud Integral”, Faculty of Dentistry and Postgraduate School, Universidad Nacional Federico Villarreal, Lima 15001, Peru; mladera@unfv.edu.pe; 3 Faculdade Do Centro Oeste Paulista, Bauru 17012, Brazil; fredy.solis@facop.com.br; 4 Faculty of Stomatology, Universidad Inca Garcilaso de la Vega, Lima 15084, Peru; luiscervantesganoza@outlook.com

**Keywords:** biosafety against COVID-19, dentistry, infection control, risk of contagion, personal protective equipment

## Abstract

Aim: Biosafety is a set of preventive measures aimed at controlling risk factors arising from biological, physical, and/or chemical agents. This topic is particularly important in the dental field since saliva is the main biological agent of the transmission of coronavirus. The present study aimed to determine the factors associated with the level of knowledge about biosafety against COVID-19 in Peruvian dentistry students. Materials and Methods: The present observational, cross-sectional, and analytical study evaluated 312 Peruvian dentistry students. A validated 20-question questionnaire was used to measure the level of knowledge. The nonparametric Mann–Whitney U and Kruskal–Wallis tests were used to compare levels of knowledge between categories of each variable. A logit model was used to evaluate associated factors such as sex, age, marital status, place of origin, academic year of study, being in the academic upper third, history of COVID-19, and living with vulnerable family members. A significance level of *p* < 0.05 was considered. Results: 36.2%, 31.4%, and 32.4% presented poor, fair, and good knowledge levels, respectively. Students under 25 years of age were 64% less likely to pass the biosafety against COVID-19 questionnaire than students 25 years of age and older (OR = 0.36; CI: 0.20–0.66). Students in the academic upper third were nine times more likely to pass the test than other students (OR = 9.38; CI: 4.61–19.07). Finally, third-year students were 52% less likely to pass the exam than fifth-year students (OR = 0.48; CI: 0.28–0.83). Conclusion: Only a minority of dentistry students had a good level of knowledge about biosafety against COVID-19. Younger and less educated students were more likely to fail the questionnaire. On the other hand, those students with outstanding academic performance were more likely to pass the questionnaire.

## 1. Introduction

In December 2019, health authorities in Wuhan, China, identified a cluster of pneumonia cases with unknown etiology linked to people attending the seafood market in southern China [1,2,3]. The first epidemiological studies carried out in this location showed that the disease was progressing rapidly and acted more aggressively in older adults. On 2 March 2020, an overall case fatality rate of 3.4% was reached. The 2019 coronavirus disease outbreak (COVID-19) is caused by a type 2 coronavirus that can cause severe acute respiratory syndrome (SARS-CoV-2). The World Health Organization (WHO) declared this nosological entity a pandemic on 11 March 2020 [3,4].

The incubation period of SARS-CoV-2 is 5 to 6 days and can be extended up to 14 days. During this period, patients remain under medical observation and undergo mandatory social isolation. It is currently known that transmission of SARS-CoV-2 occurs mainly by saliva micro droplets expelled as aerosol by the carrier when talking, coughing, or sneezing [4,5]. Some reports have shown that this virus can be suspended in the air as an aerosol for up to 4 h. This constitutes a risk factor in closed environments such as dental offices, clinics, or hospitals. The main clinical manifestations of this disease include: fever, fatigue, cough, expectoration, dyspnea, sore throat, headache, anosmia, dysgeusia, among others [5,6,7]. During the development of this research, Peru was in the second wave of COVID-19 infection with a predominance of the omicron variant SARS-CoV-2, which was distinguished by its high contagiousness and infectivity, characterized by high respiratory symptomatology, lower frequency of pulmonary involvement, lower risk of hospitalization, admission to intensive care and requirement of mechanical ventilation, headache, fatigue, sore throat, and nasal congestion [8,9,10].

Saliva is the main biological agent in the transmission of coronavirus. Therefore, in the dental field, protective measures against this vector are important since dentists are in direct contact with organic fluids that put them at constant risk of being infected and being a source of cross-infection for their patients and family members. This situation would be even more worrying if the dentist lives with vulnerable people in the family circle [11,12,13]. It is vital that dental professionals and students in their final years of dentistry school have adequate knowledge of the biosecurity measures that should be adopted before, during, and after a clinical procedure due to the constant risk of caring for a patient infected with COVID-19 [13,14].

Biosafety is a set of preventive measures aimed at controlling risk factors arising from biological, physical, and/or chemical agents. The WHO has defined it as a set of precautions, techniques, and procedures to protect the integrity of all health equipment. These should be universal since it is a fundamental principle that all patients and their fluids should be considered as potentially infectious [14,15,16]. Therefore, the correct use of protective barriers, means of disposal of contaminated material, and risk assessment are of utmost importance [17,18].

The use of personal protective equipment by the dentist against coronavirus is essential. This equipment includes protective eyewear, masks (type N95, FFP2, or FFP3), overalls, boots, and gloves [19]. Likewise, to reduce the risk of cross-infection, it is essential to use disinfectants in the fomites after each care. The WHO recommends the use of 0.5% sodium hypochlorite or 70% ethanol since it is known that SARS-CoV-2 can be stable on surfaces such as copper for up to 4 h, cardboard and stainless steel for 24 h, and plastic for 72 h [19,20,21].

Therefore, it is vitally important that dentistry students in preclinical and clinical courses have adequate knowledge of biosafety so that they can apply it in the treatment of patients with a potential risk of coronavirus infection. In addition, significant differences in the spread of COVID-19 between age groups were reported in India [22]. Another investigation in the same country reported that people aged ≥30 years were 78% more likely to have good knowledge compared to those <30 years [23]. Furthermore, in Ecuador, dental students were reported to have significantly higher knowledge of COVID-19 compared to students in other health professions; it was also found that the higher the academic semester, the higher the knowledge [24]. It was also reported in China that students older than 25 years had a good level of knowledge of COVID-19 [25]. On the other hand, a study in Vietnam reported that COVID-19 knowledge scores were positively correlated with the student’s age and year of study [26]. The present study aimed to determine the factors associated with the level of knowledge about biosafety against COVID-19 in Peruvian dentistry students.

## 2. Materials and Methods

### 2.1. Study Design

This prospective, analytical, observational, cross-sectional study was written according to the STrengthening the Reporting of OBservational studies in Epidemiology (STROBE) guidelines for observational studies [27] and was conducted from February to June 2022 at the School of Dentistry of the Universidad Privada San Juan Bautista (UPSJB) based in the Peruvian capital (Lima) and a branch in a Peruvian province (Ica).

### 2.2. Population and Selection of Participants

The study population consisted of 322 UPSJB dentistry students (121 students in the 3rd year of study, 111 students in the 4th year of study, and 90 students in the 5th year of study). No sample size calculation was required when working with the entire population. Participants totaled 312 considering the inclusion and exclusion criteria.

Inclusion criteria:

- Dentistry students enrolled in the 2022–1 semester;
- Dentistry students in their 3rd year, 4th year, and 5th year of their professional career (since only they attended preclinical and clinical courses at UPSJB. The 1st- and 2nd-year students only attended basic courses that did not include the topic of the present study);
- Dentistry students who voluntarily gave informed consent.

Exclusion criteria:

- Dentistry students who did not complete the entire questionnaire.

### 2.3. Variables

The present study considered as dependent variable the level of knowledge about biosafety against COVID-19, as independent variables the sex and age [26,27,28,29], and as possible confounding variables the academic year of study [30,31], marital status [32], place of origin [33,34], history of COVID-19 [35], being in the academic upper third, and living with vulnerable family members.

### 2.4. Validation of Instrument

A questionnaire of 20 closed multiple-choice questions [36] was validated by four judges with experience in public health and dental research. These experts evaluated the relevance, timeliness, pertinence, objectivity, methodology, and clarity of the instrument, obtaining an acceptable Aiken’s V (V = 0.89; CI: 0.85–0.91). The score for each correct answer was 1 point and for each incorrect answer 0 points. The total score was categorized from 0 to 10 points as poor, from 11 to 13 points as fair, and from 14 to 20 points as good [37,38].

Three dimensions were identified: D1 (Risk of contagion) (Q1, Q3, Q5, Q7, Q11, Q15, and Q18), D2 (Infection control measure) (Q2, Q4, Q9, Q10, Q12, and Q20), and D3 (Personal protective equipment) (Q6, Q8, Q13, Q14, Q16, Q17, and Q19), according to principal component factor analysis with Varimax rotation. In addition, the item–item correlation determinant, Bartlett’s test of sphericity, and the Kaiser–Mayer–Olkin (KMO) measure indicated values of *p* = 0.002, *p* < 0.001, and 0.766, respectively, all being acceptable values [39].

Cronbach’s alpha was used to determine the internal consistency of the instrument, obtaining an (α) of 0.72 (95% CI: 0.67–0.76), which is of acceptable reliability. To evaluate the reproducibility of the instrument, 30 randomly selected students were surveyed over a period of 7 days, at two different times and altering the order of questions to avoid recall bias. The intraclass correlation coefficient (ICC) of the scores obtained was acceptable at 0.91 (95% CI: 0.81–0.96).

### 2.5. Procedure

The questionnaire was created in the virtual platform Google Classroom^®^ and was shared through a web link directed to each student’s institutional email. In case they did not respond, the invitation was re-shared to their personal email or WhatsApp^®^. The invitation was in the charge of the principal investigator (J.S.P), who directed the invitation from his own institutional email or from his own WhatsApp^®^. When the students entered the web link, it immediately directed them to the informed consent form with the data of the principal investigator (full name, email, telephone, and university of origin) and the institutional email of the ethics committee. If the student consented, the system directed him/her to the next page where the questionnaire was located, with instructions on how to complete it. Participants had the full right to decline the invitation or not to complete the questionnaire if they wished. Only the principal investigator had access to the data and, to ensure the confidentiality of the data, they were stored in a portable digital device with a password. Only one complete response per student was accepted. To avoid repetition of responses, the virtual questionnaire was configured to allow only one response per associated email. In addition, they were asked to enter the initials of their first and last name along with their age (for example: JSP28) to filter out repetitions in case someone accessed the web link from two different e-mail addresses. The invitees did not receive any incentive for their participation and had access from 1 February to 30 June 2022.

### 2.6. Data Analysis

The data were analyzed with the Stata statistical package (College Station, TX, USA) version 17.0. For the descriptive analysis of the qualitative variables, absolute and relative frequencies were used. For the quantitative variable age, the mean was used as a measure of central tendency and the standard deviation as a measure of dispersion. For the comparison of ordinal variables, the nonparametric Mann–Whitney U test was used to compare two categories and the Kruskal–Wallis test was used to compare more than two categories. To establish the association of the independent variables with the questionnaire items, Pearson’s chi-square test was used with Fisher’s exact test for expected values less than 5. The latter was used to verify whether the distribution of the observed response was random or significantly associated with an independent variable [40]. For the multivariable analysis, the risk factors were evaluated under a logistic regression model (logit model) using odds ratio (OR) with the stepwise technique, evaluating statistical assumptions such as independent observations, sufficient sample size according to the number of independent variables, absence of multicollinearity, and goodness of fit in the model [41,42]. The significance level considered was *p* < 0.05. However, *p*-values are not adjusted for multiple testing and should only be interpreted exploratorily.

### 2.7. Ethical Aspects

The present study respected the bioethical principles of the Declaration of Helsinki related to respect, freedom, nonmaleficence, and confidentiality [43]. In addition, it had the approval of an Institutional Research Ethics Committee of the UPSJB with resolution No. 45-2022-CIEI-UPSJB and dated 17 January 2022. In addition, on the first page of the virtual questionnaire, students were asked to give voluntary informed consent.

## 3. Results

The response rate of the participants was 96.9%. The average age of the 312 dentistry students who submitted a completed survey was 24.0 ± 5.8 years. The age group under 25 years was the most abundant with 69.9% of the total, while the female gender was the most frequent with 59.6% of the total. In addition, unmarried students accounted for 84% of the total. The 32.4% of the students according to their academic performance were from the upper third. The highest percentage of students were from the third year with 37.5% of the total. Finally, 59.6% of the students reported no history of COVID-19 and 59.9% reported not living with vulnerable persons (Table 1).

**Figure 1:**
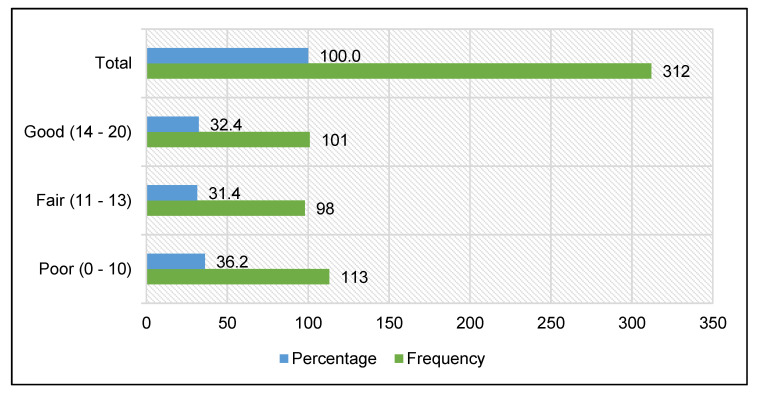
Frequency of the level of knowledge about biosafety against COVID-19 in dentistry students from a Peruvian university.

Of the 312 dentistry students surveyed, 36.2% (CI: 27.3–45.0%) had a poor level of knowledge about biosafety against COVID-19, 31.4% (CI: 22.2–40.6%) had a fair level of knowledge, and 32.4% (CI: 23.2–41.5%) had a good level of knowledge (Figure 1).

When comparing the level of knowledge about biosafety against COVID-19 in dentistry students according to their sociodemographic factors, significant differences were obtained between students under 25 years of age and those 25 years of age and older (*p* < 0.001). Significant differences were also observed between unmarried and married students (*p* = 0.025) and between students in the upper academic third and the others (*p* < 0.001). Regarding the academic year of study, significant differences (*p* = 0.002) were observed in at least two groups, so when multiple comparisons were made, only third- and fifth-year students showed significant differences (*p* = 0.005). There were also significant differences between students from the province and those from the capital (*p* = 0.009). In addition, significant differences were observed between those who reported a history of COVID-19 and those who did not (*p* = 0.014). Finally, there were significant differences between those who lived with vulnerable people and those who did not (*p* = 0.027) (Table 2).

Regarding knowledge about the risk of contagion by COVID-19, statistically significant associations were obtained between age group and Q1, Q5, and Q7 (*p* = 0.001, *p* = 0.025, and *p* = 0.004, respectively). Sex was significantly associated with Q15 (*p* = 0.009). Marital status was significantly associated with Q1, Q7, and Q11 (*p* = 0.009, *p* = 0.016, and *p* = 0.003, respectively). Belonging to the academic upper third was significantly associated with Q1, Q3, Q7, Q11, and Q18 (*p* < 0.001, *p* = 0.005, *p* < 0.001, *p* = 0.026, and *p* = 0.014, respectively). Academic year of study was significantly associated with Q1, Q5, and Q7 (*p* = 0.002, *p* = 0.011, and *p* = 0.015, respectively). Place of origin was significantly associated with Q1 (*p* = 0.012). Finally, having a history of COVID-19 was significantly associated with Q1 and Q7 (*p* = 0.038 and *p* = 0.047, respectively) (Table 3).

Regarding knowledge about infection control measures against COVID-19, significant association was observed with Q2 and Q20 (*p* = 0.004 and *p* = 0.003, respectively). Sex was significantly associated with Q2 (*p* = 0.011). Marital status was significantly associated with Q20 (*p* = 0.009). Belonging to the academic upper third was significantly associated with Q9 and Q20 (*p* = 0.004 and *p* < 0.001, respectively). Academic year of study was significantly associated with Q2, Q12, and Q20 (*p* < 0.001, *p* = 0.006, and *p* = 0.003, respectively). Place of origin was significantly associated with Q2, Q9, and Q20 (*p* = 0.007, *p* = 0.026, and *p* = 0.015). Having a history of COVID-19 was significantly associated with Q2 (*p* < 0.001). Finally, living with people vulnerable to COVID-19 was significantly associated with Q2 and Q4 (*p* = 0.002 and *p* = 0.049, respectively) (Table 4).

Regarding knowledge about personal protective equipment against COVID-19, there was a significant association of age group and marital status with Q17 (*p* = 0.016 and *p* = 0.013, respectively). Belonging to the upper academic third was significantly associated with Q6, Q16, and Q17 (*p* < 0.001, *p* < 0.001, and *p* = 0.036, respectively). Academic year of study was significantly associated with Q17 (*p* = 0.004). Place of origin was significantly associated with Q19 (*p* = 0.012). Finally, having a history of COVID-19 was significantly associated with Q8 (*p* = 0.007) (Table 5).

For the multivariable analysis, age group and sex were considered as independent variables. The intervening variables were marital status, belonging to the upper academic third, academic year of study, place of origin, having a history of COVID-19, and living with vulnerable people. The dependent variable was knowledge about biosafety against COVID-19 (Pass = 1 [11–20 points]/Fail = 0 [0–10 points]). Age group of less than 25 years (*p* = 0.013) and belonging to the academic upper third (*p* < 0.001) were significant in the crude logistic regression model. However, after performing the adjusted model with the stepwise technique, it could be observed that students under 25 years of age were 64% less likely to pass the questionnaire of knowledge about biosafety against COVID-19 compared to students aged 25 years and older (OR = 0.36; CI: 0.20–0.66). In addition, students belonging to the academic upper third were nine times more likely to pass the questionnaire than the other students (OR = 9.38; CI: 4.61–19.07). Finally, third-year students were 52% less likely to pass the questionnaire of knowledge about biosafety against COVID-19 than fifth-year students (OR = 0.48; CI: 0.28–0.83) (Table 6).

## 4. Discussion

The COVID-19 pandemic has caused changes in the labor, social, health, economic, and educational spheres [44,45]. Academic teaching had to migrate to the virtual modality [46]. Health sciences careers, including dentistry, given their high risk of contagion and cross-infection due to the characteristics of clinical procedures (spread of contaminated aerosols) [31,40,47,48], had to adapt to this type of online teaching with the expectation of gradually resuming clinical care to patients and training in manual skills as a crucial part of the learning process. It is necessary that students are prepared in biosafety protocols that guarantee an adequate performance in the clinical area. The present study aimed to determine the factors associated with the level of knowledge about biosafety against COVID-19 in dentistry students from a Peruvian university.

In the present study, 36.2% of the students surveyed had a poor level of knowledge about biosafety against COVID-19, 31.4% showed a fair level of knowledge, and 32.4% showed a good level of knowledge. These results differ from Umeizudike et al. [30], who reported that approximately 50% of their student respondents had inadequate knowledge. This discrepancy may be due to the fact that Umeizudike et al. administered their survey before mid-2020 when infodemia was widely disseminated in social networks and broadcast media. Reliable information on biosecurity was not yet available. Training on the subject was limited so that accredited knowledge about COVID-19 was still deficient. The adequate information that did exist was not well-diffused. All these circumstances during that period could have influenced the poor level of knowledge presented by the students [31,45,49,50].

The present study showed that third-year students were 52% less likely to pass the questionnaire of knowledge about biosafety against COVID-19 than fifth-year students. These results are in agreement with Umeizudike et al. [30] and Fernandez et al. [31], who found that students in higher years of study had better levels of knowledge than those in lower years. This may be due to the fact that students acquire new theoretical knowledge, greater awareness, and more clinical experience as their curriculum progresses, which would improve their level of knowledge [30,31]. Moreover, students in their last year could have a better understanding of the information related to COVID-19 due to their exposure to high-level learning and information received in their theoretical courses and practical exposures. In addition, students in their last year (5th year) received extra training on COVID-19 prevention as they were entering their hospital internship [51].

Regarding age, the present study found that students younger than 25 years were 64% less likely to pass the questionnaire of knowledge about biosafety against COVID-19. These results are similar to that reported by Zhang et al. [25], who found that one of the predictors of good knowledge about COVID-19 was being a student aged ≥25 years. Furthermore, Doan et al. [26] also indicated that the age of the students was a significant factor associated with the level of knowledge, as older students were more likely to have higher scores. This could be understood in the context that, as the years of study pass, students are able to receive more career-related training and gain more clinical experience, which can contribute to academic enrichment. Older students also attach greater importance to collaborative learning and personal development, finding it edifying and pleasurable to share knowledge among peers [25].

Among the novelties of the present study, it was found that students belonging to the academic upper third were nine times more likely to pass the questionnaire than all other students. These results may be due to the fact that these students are accustomed to competitiveness and the desire for constant improvement. Normally, these students seek to obtain certain academic benefits that are only granted to the first places (access to educational scholarships and complementary training) [52,53]. Perhaps when faced with a concern that compromises public health, they may feel extra motivation to carry out a greater search for information on this disease, resulting in a greater sense of responsibility in the face of a generalized problem and seeking to provide solutions that contribute to the development of their country [54,55,56]. All this contributed to these students being able to perform a greater search for information about COVID-19, acquiring a higher level of knowledge about this topic than the average student, despite the fact that both were under the same curricular plan [57,58,59].

In view of the impact that the COVID-19 pandemic has had on dental professionals in their clinical, administrative, and cultural practice, it is imperative to reinforce the knowledge of biosecurity protocols from the student stages in order to ensure that they can better cope with future health emergencies, reducing the levels of stress that could be generated by patient care at such times [60]. Moreover, although dental students are not yet professionals, they perform practical activities in the clinical areas of their educational institutions in order to acquire and develop their manual skills and competencies. For this reason, they are also exposed to the risks inherent to the profession [61,62]. Therefore, assessing the knowledge of students about biosafety measures to be adopted in dental clinical care can provide important evidence to support and strengthen the planning and implementation of educational programs that will prevent cross-contamination or possible contagion, safeguarding the lives of students, teachers, and patients [31].

Among the limitations of the present study, we can mention that it was not possible to survey students in person because the educational sector was in the process of adapting to on-site attendance, nor was it possible to follow up on knowledge about biosafety pre-pandemic, during the pandemic, and post-pandemic in order to evaluate the variation and durability of this knowledge over time. Finally, another limitation was that only dentistry students from a single university were included, but with campuses in the capital city and a Peruvian province, since the aim was to control the curricular plan variable. While recognizing that these results cannot be extrapolated to the whole country, they can be a starting point for future scopes of this line of research to identify knowledge gaps and take appropriate measures to plan and reinforce COVID-19 biosafety training and prevent cross-infection through professional practice in dentistry [63].

Taking into account the results obtained, it is recommended that academic managers include topics about the mitigation of infection spread, including COVID-19, in their curricular plans, with special emphasis on students in lower academic years and lower academic performance in order to improve biosafety in laboratory and clinical environments. In this way, long-term awareness could be created with appropriate protection protocols to minimize the morbimortality of students, teachers, and patients in future situations of similar public health crises [31,51,64]. It is also advisable to raise awareness among students and authorities on the importance of university outreach to the community, with constant updates and training on biosafety issues that allow citizens to become literate in order to mitigate the spread of the virus. In addition, it is recommended to replicate this study in other social realities and identify possible influencing factors on the level of the knowledge of students about biosafety, both at the undergraduate and graduate levels, considering other possible confounding variables such as socioeconomic level, type of school of origin, having children, having lost family members due to the pandemic, family history of COVID-19, among others. Finally, it is recommended to design longitudinal studies and verify whether knowledge on this subject is consolidated over time and whether it is reflected in good practices.

## 5. Conclusions

Recognizing the limitations of this cross-sectional study, it can be concluded that only a minority of dentistry students had a good level of knowledge about biosafety against COVID-19. Younger students and those with fewer years of study had a higher risk of failing the questionnaire on this topic. On the other hand, students with outstanding academic performance were more likely to pass the questionnaire. It is important to reinforce knowledge about biosafety during the professional training of dentists in order to reduce the risks of contamination and cross-infection during patient care.

## Figures and Tables

**Table 1:** Sociodemographic characteristics of dentistry students from a Peruvian university.

Variable	Category	Frequency	Percentage
**Age group**	<25 years	218	69.9
	≥25 years	94	30.1
**Sex**	Male	126	40.4
	Female	186	59.6
**Marital status**	Unmarried	262	84.0
	Married	50	16.0
**Academic upper third**	Yes	101	32.4
	No	211	67.6
**Academic year of study**	3rd year	117	37.5
	4th year	108	34.6
	5th year	87	27.9
**Place of origin**	Province	117	37.5
	Capital	195	62.5
**History of COVID-19**	Yes	126	40.4
	No	186	59.6
**Living with vulnerable people**	Yes	125	40.1
	No	187	59.9
**Age**	**Mean**	**SD**	
	24.0	5.8	

SD: Standard deviation.

**Table 2:** Comparison of the level of knowledge about biosafety against COVID-19 in dentistry students according to their sociodemographic factors.

Variable	Category	Level of Knowledge					
		Poor = 1	Fair = 2	Good = 3	Median	IQR	*p* *
		f (%)	f (%)	f (%)			
**Age group**	<25 years	92 (29.5)	69 (22.1)	57 (18.3)	2	2	<0.001 *
	≥25 years	21 (6.7)	29 (9.3)	44 (14.1)	2	1	
**Sex**	Male	49 (15.7)	43 (13.8)	34 (10.9)	2	2	0.162
	Female	64 (20.5)	55 (17.6)	67 (21.5)	2	2	
**Marital status**	Unmarried	103 (33.0)	78 (25.0)	81 (26.0)	2	2	0.025 *
	Married	10 (3.2)	20 (6.4)	20 (6.4)	2	1	
**Academic upper third**	Yes	11 (3.5)	45 (14.4)	45 (14.4)	2	1	<0.001 *
	No	102 (32.7)	53 (17.0)	56 (17.9)	2	2	
**Academic year of study**	3rd year	55 (17.6)	35 (11.2)	27 (8.7)	2 ^A^	1	0.002 *
	4th year	34 (10.9)	38 (12.2)	36 (11.5)	2 ^A,B^	2	
	5th year	24 (7.7)	25 (8.0)	38 (12.2)	2 ^B^	2	
**Place of origin**	Province	34 (10.9)	35 (11.2)	48 (15.4)	2	2	0.009 *
	Capital	79 (25.3)	63 (20.2)	53 (17.0)	2	2	
**History of COVID-19**	Yes	41 (13.1)	31 (9.9)	54 (17.3)	2	2	0.014 *
	No	72 (23.1)	67 (21.5)	47 (15.1)	2	2	
**Living with vulnerable people**	Yes	36 (11.5)	42 (13.5)	47 (15.1)	2	2	0.027 *
	No	77 (24.7)	56 (17.9)	54 (17.3)	2	2	

* Based on Mann–Whitney U, (*p* < 0.05, significant differences). ^A,B^ Different letters indicated significant differences between mean ranks in the same column as post hoc of Kruskal–Wallis test.

**Table 3:** Knowledge of dentistry students about risk of contagion.

Questions	Do Not Know	Know	Age Group	Sex	Marital Status	Academic Upper Third	Academic Year of Study	Place of Origin	History of COVID-19	Living with Vulnerable People
	f (%)	f (%)	p *	p *	p *	p *	p *	p *	p *	p *
**Q1.** At what level of risk are dentists against COVID-19?	146 (46.8)	166 (53.2)	0.001 *	0.493	0.009 *	<0.001 *	0.002 *	0.012 *	0.038 *	0.133
**Q3.** What are the risk factors for COVID-19?	91 (29.2)	221 (70.8)	0.354	0.751	0.843	0.005 *	0.307	0.630	0.409	0.257
**Q5.** During the COVID-19 pandemic, do invasive dental treatments increase the risk of infection?	153 (49.0)	159 (51.0)	0.025 *	0.229	0.277	0.273	0.011 *	0.748	0.382	0.092
**Q7.** Is it better NOT to treat dental emergencies during a high COVID-19 infection curve?	155 (49.7)	157 (50.3)	0.004 *	0.407	0.016 *	<0.001 *	0.015 *	0.057	0.047 *	0.344
**Q11.** Should a minimum distance of 1 m be maintained in the waiting room?	206 (66.0)	106 (34.0)	0.305	0.097	0.003 *	0.026 *	0.310	0.666	0.437	0.709
**Q15.** What are the routes of transmission for COVID-19?	84 (26.9)	228 (73.1)	0.231	0.009 *	0.218	0.384	0.852	0.356	0.447	0.258
**Q18.** What are the most frequent sources of cross-contamination or cross-infection in the dental practice?	107 (34.3)	205 (65.7)	0.271	0.959	0.485	0.014 *	0.591	0.441	0.848	0.346

* Based on Pearson’s chi-square (* *p* < 0.05, significant association). For expected values less than 5, Fisher’s exact test was used (* *p* < 0.05, significant association).

**Table 4:** Knowledge of dentistry students about infection control measures.

Questions	Do Not Know	Know	Age Group	Sex	Marital Status	Academic Upper Third	Academic Year of Study	Place of Origin	History of COVID-19	Living with Vulnerable People
	f (%)	f (%)	p *	p *	p *	p *	p *	p *	p *	p *
**Q2.** What chemical substance can be used as a clothing disinfecting agent?	215 (68.9)	97 (31.1)	0.004 *	0.011 *	0.137	0.229	<0.001 *	0.007 *	<0.001 *	0.002 *
**Q4.** What is the right temperature and time to sterilize instruments in dry heat?	276 (88.5)	36 (11.5)	0.953	0.846	0.911	0.610	0.817	0.583	0.598	0.049 *
**Q9.** What alcohol concentration is suitable for surface disinfection against COVID-19?	78 (25.0)	234 (75.0)	0.064	0.230	0.593	0.004 *	0.514	0.026 *	0.143	0.095
**Q10.** Hand washing is performed only after each clinical procedure.	27 (8.7)	285 (91.3)	0.349	0.969	0.858	0.750	0.657	0.107	0.969	0.940
**Q12.** Timing of hand washing to decrease COVID-19 viral load.	225 (72.1)	87 (27.9)	0.828	0.320	0.175	0.301	0.006 *	0.922	0.211	0.185
**Q20.** Which mouthwash is indicated to the patient in his daily oral hygiene to reduce the COVID-19 viral load?	153 (49.0)	159 (51.0)	0.003 *	0.520	0.009 *	<0.001 *	0.003 *	0.015 *	0.117	0.446

* Based on Pearson’s chi-square (* *p* < 0.05, significant association). For expected values less than 5, Fisher’s exact test was used (* *p* < 0.05, significant association).

**Table 5:** Knowledge of dentistry students about personal protective equipment.

Questions	Do Not Know	Know	Age Group	Sex	Marital Status	Academic Upper Third	Academic Year of Study	Place of Origin	History of COVID-19	Living with Vulnerable People
	f (%)	f (%)	p *	p *	p *	p *	p *	p *	p *	p *
**Q6.** Should personal protective equipment (PPE) only be used for symptomatic patients?	46 (14.7)	266 (85.3)	0.765	0.150	0.871	<0.001 *	0.417	0.934	0.608	0.429
**Q8.** Should protective eyewear be sterilized in an autoclave after use?	54 (17.3)	258 (82.7)	0.930	0.330	0.790	0.080	0.505	0.938	0.007 *	0.618
**Q13.** What is the sequence for putting on PPE before caring for patients in order to avoid COVID-19?	151 (48.4)	161 (51.6)	0.266	0.165	0.805	0.096	0.852	0.884	0.486	0.564
**Q14.** Should the cap cover all the hair and prevent it from falling towards the front and sides of the face?	47 (15.1)	265 (84.9)	0.276	0.105	0.509	0.277	0.546	0.902	0.108	0.483
**Q16.** When treating several patients, can the conventional surgical mask be used for a maximum of one day?	177 (56.7)	135 (43.3)	0.867	0.199	0.909	<0.001 *	0.538	0.118	0.297	0.252
**Q17.** Which mask is recommended for use in dental care against COVID-19?	89 (28.5)	223 (71.5)	0.016 *	0.300	0.013 *	0.036 *	0.004 *	0.099	0.810	0.051
**Q19.** What is the sequence for removing PPE after taking care of a patient in order to avoid COVID-19?	146 (46.8)	166 (53.2)	0.324	0.482	0.902	0.429	0.232	0.012 *	0.650	0.564

* Based on Pearson’s chi-square (* *p* < 0.05, significant association). For expected values less than 5, Fisher’s exact test was used (* *p* < 0.05, significant association).

**Table 6:** Multivariable analysis of knowledge about biosafety against COVID-19 according to associated factors of dentistry students.

Variable	Category	Crude Model					Adjusted Model				
		β	OR	95% CI		*p*	β	OR	95% CI		p *
				LL	UL				LL	UL	
**Age group**	<25 years	−0.86	0.42	0.21	0.83	0.013	−1.02	0.36	0.20	0.66	0.001 *
	≥25 years		*Ref.*					*Ref.*			
**Sex**	Male	−0.28	0.76	0.44	1.32	0.326					
	Female		*Ref.*								
**Marital status**	Unmarried	−0.50	0.61	0.24	1.50	0.280					
	Married		*Ref.*								
**Academic upper third**	Yes	2.28	9.76	4.74	20.08	<0.001	2.24	9.38	4.61	19.07	<0.001 *
	No		*Ref.*					*Ref.*			
**Academic year of study**	3rd year	−0.57	0.56	0.28	1.15	0.116	−0.73	0.48	0.28	0.83	0.008 *
	4th year	0.03	1.03	0.51	2.10	0.930					
	5th year		*Ref.*					*Ref.*			
**Place of origin**	Province	0.30	1.36	0.75	2.43	0.309					
	Capital		*Ref.*								
**History of COVID-19**	Yes	−0.03	0.97	0.56	1.68	0.919					
	No		*Ref.*								
**Living with vulnerable people**	Yes	0.52	1.68	0.97	2.91	0.065					
	No		*Ref.*								
***Model constant***		1.10	3.01	1.04	8.70	0.042	1.04	2.84	1.67	4.83	<0.001

* Adjusted logit model for all variables that resulted in a *p*-value < 0.05 in the crude model. β: coefficient of determination; OR = odds ratio; and 95% CI = 95% confidence interval. For the adjusted model of knowledge about biosafety against COVID-19, the Pseudo R^2^ = 0.171, *p* < 0.001 (significant for the omnibus test of the model coefficient).

## Data Availability

The data presented in this study are available upon request from the corresponding author (cesarcayorojas@gmail.com).
